# Predictive role of circulatory HMGB1 in postoperative acute exacerbation of interstitial lung disease in lung cancer patients

**DOI:** 10.1038/s41598-021-89663-w

**Published:** 2021-05-12

**Authors:** Kakuhiro Yamaguchi, Satoshi Nakao, Hiroshi Iwamoto, Atsushi Kagimoto, Yoshinori Handa, Shinjiro Sakamoto, Yasushi Horimasu, Takeshi Masuda, Takahiro Mimae, Shintaro Miyamoto, Taku Nakashima, Yasuhiro Tsutani, Kazunori Fujitaka, Yoshihiro Miyata, Hironobu Hamada, Morihito Okada, Noboru Hattori

**Affiliations:** 1 Department of Molecular and Internal Medicine, Graduate School of Biomedical and Health Sciences, Hiroshima University, 1-2-3 Kasumi, Minami-ku, Hiroshima, 734-8551 Japan; 2 Department of Surgical Oncology, Division of Radiation Biology and Medicine, Graduate School of Biomedical & Health Sciences, Hiroshima University, Hiroshima, Japan; 3 Department of Physical Analysis and Therapeutic Sciences, Graduate School of Biomedical and Health Sciences, Hiroshima University, Hiroshima, Japan

**Keywords:** Predictive markers, Cancer therapy, Lung cancer

## Abstract

Postoperative acute exacerbation of interstitial lung disease (AE-ILD) can be fatal in patients with lung cancer concomitant with ILD. We aimed to elucidate the predictive potential of high-mobility group box 1 (HMGB1), which is associated with the development and severity of lung injury, for evaluating the risk of this complication. We included 152 patients with lung cancer and ILD who underwent radical surgery between January 2011 and August 2019. We evaluated the preoperative levels of serum HMGB1 and its predictive potential for postoperative AE-ILD. Postoperative AE-ILD developed in 17 patients. Serum levels of HMGB1 were significantly higher in patients with postoperative AE-ILD than in those without (median [interquartile range]: 5.39 [3.29–11.70] ng/mL vs. 3.55 [2.07–5.62] ng/mL). Univariate and multivariate logistic regression analyses revealed that higher HMGB1 levels were significantly associated with the development of postoperative AE-ILD in entire studied patients (n = 152). In the subgroup analysis, higher HMGB1 levels were associated with a significantly increased risk of this complication in patients who underwent lobectomy (n = 77) than in those who underwent sublobar resection (n = 75). Serum HMGB1 could be a promising marker for evaluating the risk of postoperative AE-ILD, specifically in patients who underwent lobectomy.

## Introduction

High-mobility group box 1 (HMGB1) is a nuclear protein that is involved in DNA organization and the regulation of transcription^1^. HMGB1 is released passively during cellular necrosis by almost all cells. It is also secreted actively by immune cells, including monocytes, macrophages, and dendritic cells^2^. Circulatory HMGB1 binds to pattern recognition receptors on the cell surface, such as the receptor for advanced glycation end product (RAGE) and toll-like receptor (TLR). Their interactions result in the activation of pro-inflammatory responses associated with acute lung injury^3,4^. We have previously reported that serum levels of HMGB1 are significantly higher in patients with idiopathic pulmonary fibrosis (IPF) than in healthy subjects, and its higher levels at baseline are associated with the earlier development of acute exacerbation of IPF (AE-IPF)^5^. Additionally, when AE-IPF is developed, HMGB1 levels are further increased compared to those in IPF patients without AE^5,6^. Although the trigger for AE-IPF has not been sufficiently elucidated, these data suggest that higher levels of HMGB1 play a role in the development of lung injury superimposed with interstitial lung disease (ILD), including IPF.

Postoperative AE-ILD is a fatal complication in patients with lung cancer treated with radical surgery, especially when concomitant with ILD. Previous reports have shown that postoperative AE-ILD occurs in 9.3% of patients with lung cancer and ILD and has a mortality rate of 43.9%^7^. Although lobectomy, as a standard radical treatment, is generally associated with a higher risk of postoperative AE-ILD, compared to sublobar resection^7^, the pathophysiology of this fatal adverse event is still unclear. Several studies have demonstrated that HMGB1-induced lung injury is accelerated during surgery. For example, mechanical ventilation induces elevated expression of HMGB1 in lung tissue and bronchoalveolar lavage fluid (BALF), and anti-HMGB1 antibody can ameliorate ventilation-induced lung injury^8,9^. These data led us to speculate that HMGB1 is associated with the development of postoperative AE-ILD as well as AE-IPF, and thus we hypothesize that baseline levels of circulatory HMGB1 can be a predictive blood marker of this severe complication.

This study aimed to elucidate the association between serum levels of HMGB1 and the development of postoperative AE-ILD in patients with lung cancer concomitant with ILD. First, the association of baseline levels of serum HMGB1 with the incidence rate of postoperative AE-ILD was analyzed. Second, its association was separately evaluated in the subgroups treated with lobectomy and those treated with sublobar resection. Finally, a logistic regression model was used to identify the independent risks of this severe complication.

## Results

### Patient characteristics

The subjects were 157 patients with lung cancer and ILD who underwent radical surgery and had blood samples collected before surgery. After excluding 5 patients who had another primary cancer or active autoimmune diseases, 152 patients with lung cancer and ILD were included in the analysis (Table 1). Of the 152 patients, 139 patients had idiopathic interstitial pneumonia and 13 patients had secondary ILD related to well-controlled autoimmune disease. In this study, operative time exhibited a positive correlation with bleeding volume (*r*_*s*_ = 0.640, *P* < 0.001) (Figure not shown).

**Table 1:** Baseline characteristics.

	All subjects	AE-ILD ( +)	AE-ILD (-)	*P*-value*
Subjects, n (%)	152	17 (11.2)	135 (88.8)	
Age, years	72.9 ± 7.5	71.7 ± 7.5	73.1 ± 7.5	0.487
Sex, male/female	126/26	17/0	109/26	0.045
Smoking history, pack-years	50.0 (30.0–67.5)	60.0 (39.0–95.0)	47.5 (30.0–65.6)	0.211
VC, % predicted	90.8 ± 16.2	81.9 ± 17.1	91.9 ± 15.8	0.016
FVC, % predicted	87.9 ± 15.2	81.8 ± 16.8	88.7 ± 14.8	0.076
FEV1, % predicted	87.9 ± 17.8	79.8 ± 18.2	88.9 ± 17.6	0.046
FEV1/FVC, %	75.7 ± 10.4	79.0 ± 8.1	75.3 ± 10.6	0.164
DLco, % predicted	56.5 ± 17.4	51.2 ± 21.1	57.2 ± 16.8	0.237
**ILD pattern**				0.445
UIP	48	8	40	
Probable UIP	17	2	15	
Indeterminate UIP	48	3	45	
Alternative diagnosis	39	4	35	
Preoperative steroid use, + /−	15/137	2/15	13/122	0.676
Preoperative pirfenidone use, + /−	6/146	1/16	5/130	0.664
**Histology**				0.689
Adenocarcinoma	68	6	62	
Squamous cell carcinoma	56	7	49	
Small cell carcinoma	11	2	9	
Others	17	2	15	
pStage, I/II/IIIA/IIIB	119/17/13/3	13/2/1/1	106/15/12/2	0.746
Primary tumor size, mm	24 (17–34)	33 (21–37)	22 (16–33)	0.057
**Surgical intervention**				0.474
Sublobar resection**	75	7	68	
Lobectomy	77	10	67	
Operative time, min	149 (108–196)	200 (138–252)	146 (106–181)	0.022
Bleeding volume, mL	66 (23–110)	155 (73–301)	60 (22–100)	0.003

Data are presented as mean ± standard deviation or median (interquartile range) according to their distribution.

*AE-ILD* acute exacerbation of interstitial lung disease; *DLco* diffusing capacity for carbon monoxide; *FEV1* forced expiratory volume in one second; *FVC* forced vital capacity; *ILD* interstitial lung disease; *UIP* usual interstitial pneumonia; *VC* vital capacity.

*All *P-*values were evaluated by comparing patients with and without postoperative AE-ILD using the t-test and Mann–Whitney *U* tests for normally and non-normally distributed variables, respectively, and using Pearson’s chi-squared test.

**Sublobar resection group included 41 patients treated with wedge resection and 34 patients treated with segmentectomy.

The patients with postoperative AE-ILD were male dominant and had significantly lower vital capacity (VC) (% predicted), lower forced expiratory volume in one second (FEV1) (% predicted), longer operative time, and greater bleeding volume than patients without postoperative AE-ILD (Table 1). Additionally, 1/17 patients with postoperative AE-ILD and 5/135 patients without were treated with pirfenidone before surgery. There was no association between pirfenidone and AE-ILD (*P* = 0.664). Only one patient with postoperative AE-ILD was treated with induction chemotherapy prior to surgery.

### The association of baseline concentration of HMGB1 with postoperative AE-ILD and baseline characteristics

**Figure 1:**
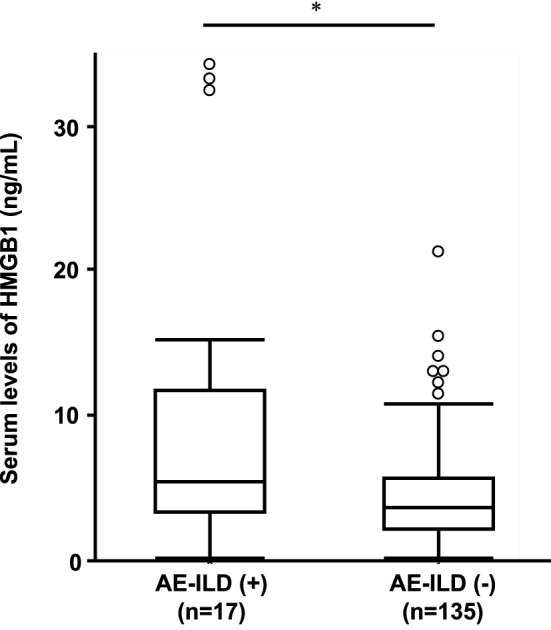
Baseline levels of serum HMGB1. Serum levels of HMGB1 in patients with postoperative acute exacerbation of interstitial lung disease (AE-ILD) were higher than those in patients without (median [IQR]: 5.39 [3.29–11.70] ng/mL vs. 3.55 [2.07–5.62] ng/mL, *P* = 0.031). Boxes represent the 25th to 75th percentiles; solid lines within the boxes show the median values; whiskers represent the 10th and 90th percentiles; the circles represent outliers. * *P* < 0.05 using the Mann–Whitney *U* test. IQR, interquartile range.

Overall, 17 patients developed postoperative AE-ILD. Serum levels of HMGB1 were significantly higher in patients with postoperative AE-ILD than in those without (median [IQR]: 5.39 [3.29–11.70] ng/mL vs. 3.55 [2.07–5.62] ng/mL, *P* = 0.031) (Fig. 1). Higher baseline levels of HMGB1 were significantly and independently associated with younger age, higher pack-year smoking history, and lower VC (Table 2).

**Table 2:** Linear regression analysis to elucidate the factors associated with preoperative levels of serum HMGB1.

Variables	*β*	*t*	*P*-value
**Univariate analysis**			
Age, years	− 0.165	− 2.04	0.043*
Sex, male	0.127	1.57	0.118
Smoking history, pack-years	0.243	3.06	0.003*
VC, %predicted	− 0.179	− 2.23	0.027*
FVC, % predicted	− 0.118	− 1.45	0.149
FEV1, % predicted	− 0.144	− 1.78	0.077
FEV1/FVC, %	0.049	0.60	0.550
DLco, % predicted	− 0.099	− 1.22	0.226
ILD pattern, UIP	0.042	0.52	0.603
Preoperative steroid use, +	− 0.008	− 0.10	0.917
pStage, I/II/III	0.017	0.20	0.838
Primary tumor size, mm	0.139	1.72	0.087
CEA, ng/mL	0.016	0.19	0.848
KL-6, U/mL	0.013	0.16	0.876
FDG uptake of primary tumor, SUV_max_	0.116	1.40	0.164
**Multivariate analysis**^**#**^			
Age, years	− 0.170	− 2.19	0.030*
Smoking history, pack-years	0.210	2.62	0.010*
VC, % predicted	− 0.164	− 2.08	0.040*

*AE-ILD* acute exacerbation; *CEA* carcinoembryonic antigen; *DLco* diffusing capacity for carbon monoxide; *FDG* fluorodeoxyglucose; *FEV1* forced expiratory volume in one second; *FVC* forced vital capacity; *HMGB1* high-mobility group box 1; *ILD* interstitial lung disease; *KL-6* Krebs von den Lungen 6; *SUV* standard uptake value; *UIP* usual interstitial pneumonia; *VC* vital capacity.

*P < 0.05 Linear regression model.

^#^Confounders with a p-value < 0.05 according to the univariate analysis, were included.

### The risk stratification of postoperative AE-ILD based on HMGB1

**Figure 2:**
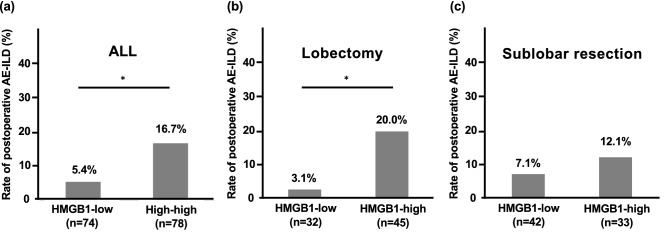
The incidence of postoperative AE-ILD based on HMGB1 and type of surgical intervention. The cut-off level for HMGB1 was 3.82 ng/mL. In the entire population, the incidence of postoperative acute exacerbation of interstitial lung disease (AE-ILD) in patients with HMGB1-high was significantly higher than that in patients with HMGB1-low **(a)**. It was also significant in the subgroup treated with lobectomy **(b)**, but not in those treated with sublobar resection **(c)**. * P < 0.05 using Pearson’s chi-squared tests.

Receiver operating characteristic (ROC) curve analysis revealed that the optimal cut-off level of serum HMGB1 for predicting postoperative AE-ILD was 3.82 ng/mL (area under the curve [AUC] 0.661, specificity 51.9%, and sensitivity 76.5%). Serum levels of HMGB1 > 3.82 ng/mL was significantly associated with a higher incidence of postoperative AE-ILD in the entire population (Fig. 2a). To elucidate the influence of surgery as the trigger for postoperative AE-ILD, the incidence was evaluated separately by the type of surgical intervention: lobectomy and sublobar resection. Subgroup analysis revealed that the association was significant in the lobectomy group, but not in the sublobar resection group (Fig. 2b,c).

Univariate logistic regression analysis revealed that lower VC, lower FEV1, longer operative time, greater bleeding volume, and higher levels of serum HMGB1 were significantly associated with increased risk of postoperative AE-ILD (Table 3a). Because of the significant correlation between operative time and bleeding volume, they were separately included in the multivariate logistic regression analysis. In the first multivariate analysis, operative time and higher levels of serum HMGB1 were significantly associated with the development of postoperative AE-ILD in the entire population (n = 152). In the subgroup analysis, higher HMGB1 significantly increased the risk of this complication in the patients who underwent lobectomy (n = 77) (Table 3b). In the second multivariate analysis including bleeding volume, both higher HMGB1 and greater bleeding volume were significantly associated with an increased risk of AE-ILD in the entire population as well as in the subgroup of patients who underwent lobectomy (Table 3c).

**Table 3:** Logistic regression analysis of the risk factors of postoperative AE-ILD.

Variables	ALL (n = 152)			Lobectomy (n = 77)			Sublobar resection (n = 75)		
	OR	95% CI	*P*-value	OR	95% CI	*P*-value	OR	95% CI	*P*-value
**(a) Univariate analysis**									
Age, years	0.975	0.911–1.045	0.475	0.949	0.865–1.041	0.266	1.025	0.920–1.143	0.652
Sex, male	Not calculated			Not calculated			Not calculated		
Smoking history, pack-years	1.010	0.999–1.020	0.076	1.013	0.999–1.026	0.058	1.003	0.984–1.022	0.771
VC, % predicted	0.965	0.936–0.994	0.020*	0.948	0.895–1.005	0.072	0.963	0.924–1.004	0.075
FVC, % predicted	0.970	0.938–1.003	0.077	0.969	0.916–1.025	0.267	0.965	0.922–1.011	0.132
FEV1, % predicted	0.970	0.943–1.000	0.045*	0.959	0.915–1.005	0.083	0.976	0.937–1.017	0.247
FEV1/FVC, %	1.036	0.986–1.089	0.163	1.046	0.974–1.123	0.221	1.032	0.959–1.109	0.399
DLco, % predicted	0.979	0.950–1.010	0.180	0.947	0.902–0.995	0.030*	1.004	0.961–1.049	0.859
Histology, NSCLC	0.536	0.106–2.715	0.451	0.188	0.027–1.297	0.090	Not calculated		
Stage I vs. II-III	0.775	0.233–2.573	0.677	0.594	0.151–2.342	0.457	Not calculated		
Primary tumor size, mm	1.024	0.990–1.059	0.167	1.036	0.991–1.083	0.119	0.996	0.932–1.063	0.896
ILD pattern, UIP	2.111	0.760–5.863	0.157	3.786	0.960–14.933	0.057	1.212	0.251–5.851	0.811
Preoperative steroid use, +	1.251	0.257–6.089	0.781	1.130	0.122–10.50	0.915	1.452	0.152–13.87	0.746
Preoperative pirfenidone use, +	1.625	0.178–14.80	0.667	Not calculated			Not calculated		
Surgical procedure, lobectomy	1.45	0.521–4.033	0.477	–	–	–	–	–	–
Operative time, min	1.010	1.003–1.018	0.008*	1.017	1.005–1.030	0.007*	1.005	0.995–1.015	0.321
Bleeding volume, mL	1.002	1.001–1.004	0.002*	1.003	1.000–1.005	0.019*	1.004	0.999–1.009	0.131
HMGB1, ng/mL	1.141	1.050–1.239	0.002*	1.170	1.054–1.298	0.003*	0.926	0.653–1.313	0.665
**(b) Multivariate analysis including operative time**									
VC, % predicted	0.966	0.923–1.011	0.132	0.983	0.906–1.068	0.697	0.946	0.891–1.004	0.070
FEV1, % predicted	0.994	0.953–1.038	0.795	0.977	0.908–1.052	0.541	1.001	0.947–1.059	0.959
Operative time, min	1.010	1.002–1.018	0.010*	1.017	1.003–1.032	0.018*	1.008	0.998–1.019	0.119
HMGB1, ng/mL	1.119	1.023–1.224	0.014*	1.178	1.037–1.338	0.012*	0.792	0.534–1.175	0.247
**(c) Multivariate analysis including bleeding volume**									
VC, % predicted	0.961	0.913–1.011	0.125	0.979	0.896–1.070	0.640	0.932	0.867–1.002	0.055
FEV1, % predicted	0.997	0.950–1.047	0.910	0.955	0.880–1.036	0.269	1.025	0.956–1.099	0.488
Bleeding volume, mL	1.003	1.001–1.005	0.009*	1.003	1.001–1.005	0.017*	1.006	1.000–1.013	0.045*
HMGB1, ng/mL	1.114	1.023–1.215	0.014*	1.170	1.027–1.332	0.019*	0.785	0.524–1.175	0.240

*AE-ILD* acute exacerbation of interstitial lung disease; *OR* Odds ratio; *CI* confidence interval; *DLco* diffusing capacity for carbon monoxide; *FEV1* forced expiratory volume in one second; *FVC* forced vital capacity; *HMGB1* high-mobility group box 1; *ILD* interstitial lung disease; *NSCLC* non-small cell lung cancer; *SCLC* small cell lung cancer; *UIP* usual interstitial pneumonia; *VC* vital capacity.

*P < 0.05 Logistic regression analysis.

To investigate this discrepancy based on lobectomy and sublobar resection, exploratory analysis revealed that the patient subgroup treated with lobectomy had a significantly younger age, higher DLco, larger primary tumor size, longer operative time, and greater bleeding volume than those treated with sublobar resection (Table 4). Among these five factors, operative time and bleeding volume were significantly different between the patients with and without postoperative AE-ILD (Table 1).

**Table 4:** Patient characteristics based on the type of surgical intervention.

	Sublobar resection^#^	Lobectomy	*P*-value
Subjects, n (%)	75 (49.3)	77 (50.7)	
Age, years	74.4 ± 7.2	71.5 ± 7.5	0.023*
Sex, male/female	65/10	61/16	0.221
Smoking history, pack-years	46 (30.8–71.3)	50 (30.0–65.3)	0.883
VC, % predicted	88.6 ± 19.2	92.9 ± 12.4	0.105
FVC, % predicted	86.5 ± 17.6	89.3 ± 12.4	0.262
FEV1, % predicted	86.7 ± 19.6	89.0 ± 16.0	0.426
FEV1/FVC, %	76.4 ± 11.2	75.1 ± 9.5	0.431
DLco, % predicted	52.7 ± 17.8	60.2 ± 16.2	0.008*
**ILD pattern**			0.270
UIP	29	19	
Probable UIP	7	10	
Indeterminate UIP	23	25	
Alternative diagnosis	16	23	
Preoperative steroid use, + /−	8/67	7/70	0.745
Preoperative pirfenidone use, + /−	5/70	1/76	0.089
**Histology**			0.220
Adenocarcinoma	32	36	
Squamous cell carcinoma	32	24	
Small cell carcinoma	6	5	
Others	5	12	
pStage, I/II/IIIA/IIIB	65/5/4/1	54/12/9/2	0.100
Primary tumor size, mm	18 (12–28)	29 (21–39)	< 0.001*
Operative time, min	139 (77–182)	159 (124–209)	0.006*
Bleeding volume, mL	47 (15–85)	80 (41–140)	< 0.001*
Incidence of postoperative AE-ILD, n (%)	7 (9.3)	10 (13.0)	0.473

Data are presented as mean ± standard deviation or median (interquartile range) according to their distribution.

*AE-ILD* acute exacerbation of interstitial lung disease; *CEA* carcinoembryonic antigen; *DLco* diffusing capacity for carbon monoxide; *FEV1* forced expiratory volume in one second; *FVC* forced vital capacity; *ILD* interstitial lung disease; *UIP* usual interstitial pneumonia; *VC* vital capacity.

*All *P-*values were evaluated by comparing patients with and without postoperative AE-ILD using the t-test and Mann–Whitney *U* tests for normally and non-normally distributed variables and using Pearson’s chi-squared test.

^#^Sublobar resection group included 41 patients treated with wedge resection and 34 patients treated with segmentectomy.

**Figure 3:**
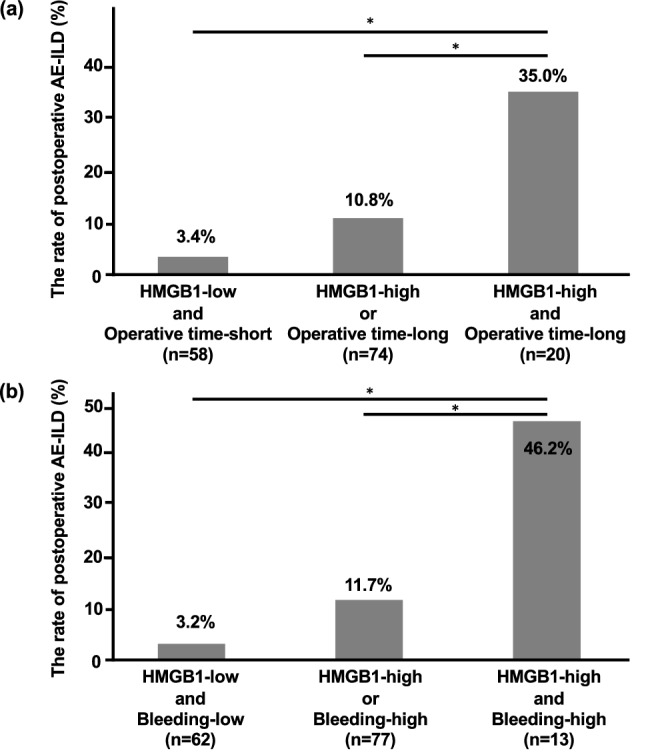
The risk stratification based on HMGB1 and factors related to surgical invasiveness. The cut-off levels of operative time and bleeding volume were 200 min and 155 mL, respectively. When HMGB1 and operative time/bleeding volume were combined, the incidence of postoperative acute exacerbation of interstitial lung disease (AE-ILD) in patients with both HMGB1-high and operative time-long/bleeding volume-high was significantly higher than that in the other two groups (**a**,**b**). *P < 0.05, using Pearson’s chi-squared tests.

Operative time longer than 200 min (AUC 0.671, specificity 58.9%, and sensitivity 80.0%) was associated with a higher incidence of this fatal complication (25.0% vs. 6.9%, *P* = 0.005). When the operative time was co-analyzed with HMGB1, combination of “higher HMGB1 and longer operative time” was associated with the highest incidence of this complication compared to those with either HMGB1-high or operative time-long and those with neither (35.0%, 10.8%, and 3.4%, respectively) (Fig. 3a). Additionally, ROC curve analysis, which was used to predict postoperative AE-ILD using HMGB1 and operative time, provided an AUC of 0.728, which is higher than that calculated by each of HMGB1 and operative time (0.661 and 0.671, respectively).

Bleeding volume greater than 155 mL (AUC 0.720; specificity 88.9%; sensitivity 52.9%) was also associated with a higher incidence of AE-ILD (32.0% vs. 7.1%, *P* < 0.001). When the bleeding volume was co-analyzed with HMGB1, a combination of “higher HMGB1 and higher bleeding volume” was associated with the highest incidence of AE-ILD (46.2%), compared to those with either HMGB1-high or bleeding volume-high (11.7%) and those with neither (3.2%) (Fig. 3b). Additionally, ROC curve analysis was used to predict postoperative AE-ILD using HMGB1 and bleeding volume, and the AUC of 0.750 was higher than that calculated by HMGB1 and operative time each (0.661 and 0.720, respectively).

## Discussion

Postoperative AE-ILD is a fatal complication, and thus, it is important to increase its predictive accuracy before surgery. This study showed that higher levels of serum HMGB1 were significantly associated with a higher incidence of postoperative AE-ILD in patients with lung cancer concomitant with ILD, especially in patients who underwent lobectomy. Additionally, the significance of HMGB1 for predicting this fatal complication was also confirmed when adjusted for lower pulmonary function parameters, which are well-known risk factors for postoperative comorbidities. No blood markers for predicting postoperative AE-ILD caused by lung resection have been reported; therefore, this study showed that HMGB1 can be a promising biomarker for predicting postoperative AE-ILD in patients with lung cancer and ILD, especially in those treated with lobectomy.

This study is the first to show that higher levels of HMGB1 were significantly associated with a higher incidence of postoperative AE-ILD in patients with lung cancer and ILD. We and others previously reported that HMGB1 levels in serum and BALF in patients with IPF were significantly elevated compared to those in healthy subjects^5,10^. Additionally, circulatory levels of HMGB1 are elevated in patients with lung cancer^11,12^. Importantly, HMGB1 itself accelerates pro-inflammatory signaling by interacting with RAGE and TLR4^3,4^, and intratracheal administration of HMGB1 induces acute lung injury^13^. This study also demonstrated that there was a negative correlation between HMGB1 and VC. These observations suggest that higher levels of circulating HMGB1 may accelerate underlying lung damage as well as indicate its severity. Thus, its higher levels can predict the development of postoperative AE-ILD.

The association of higher levels of serum HMGB1 with higher incidence of postoperative AE-ILD was significant only when the patients underwent lobectomy, which required longer operative time and greater bleeding volume, in this study. Additionally, this exploratory study showed that patients with both higher HMGB1 and longer operative time/higher bleeding volume had a significantly increased risk of postoperative AE-ILD than patients with either higher HMGB1 or longer operative time/higher bleeding volume and patients with neither. When the operative time increases, it requires a longer time for artificial ventilation, which increases the expression of HMGB1 in BALF and lung tissue in a tidal volume- and time-dependent manner^8,9^. Exposure to hyperoxia during artificial ventilation also promotes the increased expression of HMGB1 in a time-dependent manner^14^. This mechanical ventilation- and hyperoxia-induced lung injury can be attenuated by inhibition of HMGB1^8,14^. Additionally, longer operative time was correlated with greater bleeding volume, which is also reported to increase the expression of HMGB1 in the plasma and lung^15^. These observations suggest that lobectomy, which requires longer operative time, might be associated with an accelerated inflammatory response via HMGB1 secretion. Thus, lobectomy results in the development of postoperative AE-ILD, especially in patients with higher levels of preoperative HMGB1.

Lobectomy is generally performed as a standard radical treatment for lung cancer, but it is also associated with higher postoperative morbidity of complications, especially when the patient has concurrent ILD^16^. Preventing this fatal complication is crucial to improve the long-term outcomes in patients with lung cancer and ILD who underwent surgical resection. According to this study, one potential preventive strategy is that patients with higher HMGB1 would be treated with sublobar resection because it generally requires shorter operative time than lobectomy. Another potential strategy is to use HMGB1-inhibitory drugs. Some existing drugs have been reported to inhibit HMGB1-induced inflammatory signaling by decreasing HMGB1 secretion and capturing HMGB1^17^.

Another aspect of this study was to confirm the pathogenic association between AE-ILD caused by known triggers and increased HMGB1 levels. The diagnostic criteria of AE-IPF revised in 2016 have demonstrated two types of AE: idiopathic and triggered AE. Triggered AEs can be caused by surgery and drug toxicity. In line with this study showing the association between higher serum HMGB1 levels and a higher risk of postoperative AE, we have previously reported that higher levels of HMGB1 are associated with an earlier onset of cytotoxic chemotherapy-induced lung injury in patients with advanced lung cancer and ILD^11^. It should be noted that these two potential triggers, such as chemotherapy and surgery, have been reported to increase the expression of HMGB1^8,9,15,18^. These data imply that the additional release of HMGB1 by triggers of AE promotes the development of triggered AE, especially when baseline levels of HMGB1 are high. Therefore, the combination of HMGB1 with invasiveness of surgery may be able to stratify the risk of AE-ILD with higher accuracy compared to HMGB1 alone. Further investigations are needed to elucidate the utility of serum HMGB1 as a predictive biomarker and molecular target for preventing triggered AE, including postoperative AE-ILD, in patients with lung cancer and ILD.

This study has several limitations. First, this was a single-institution study, and the number of patients with postoperative AE-ILD was relatively small. These factors may substantially impact the generalizability of the study findings. Multicenter studies with a larger sample size are needed to validate the predictive value and optimal cut-off levels of HMGB1. Second, the main source of circulating HMGB1 was not identified in this study. HMGB1 may be elevated not only by the presence of ILD, but also by lung cancer. We and others have reported that HMGB1 is elevated by the presence of as well as the progression of lung cancer^11,12^. However, this study revealed a significant association between HMGB1 and VC, but not with FEV1, DLco, primary tumor size, cancer stage, and fluorodeoxyglucose uptake in cancer. Further investigations are warranted to elucidate the associations between cancer-derived HMGB1 with its magnitude and influence on lung damage, and the development of postoperative AE-ILD.

In conclusion, higher levels of circulatory HMGB1 are associated with postoperative AE-ILD in patients with lung cancer and ILD. This might be more useful in patients treated with lobectomy as a standard radical surgery.

## Methods

### Study design and population

This retrospective cohort study consecutively included lung cancer patients with ILD who collected blood samples before surgery and underwent radical surgery for lung cancer between January 2011 and August 2019 at the Hiroshima University Hospital. The type of surgical intervention was determined as follows: sublobar resection was mainly performed in patients unable to tolerate lobectomy due to poor pulmonary function. Additionally, sublobar resection was considered for small (≤ 20 mm) tumors. Wedge resection or segmentectomy was performed for peripherally or centrally located tumors, respectively. The staging of lung cancer was according to the TNM Classification of Malignant Tumors, 8th edition^19^.

This study was approved by the Ethics Committee of Hiroshima University Hospital. All research was performed in accordance with the ethical standards established by the Helsinki Declaration of 1975. To research the risk of postoperative AE-ILD, all participants provided written informed consent for obtaining and using the blood samples before surgery (Gen-38). Additionally, an opt-out method was used to inform the patients or legally authorized representatives that serum levels of HMGB1 was measured by using the already gathered samples (E-1707).

### Diagnostic criteria for ILD and postoperative AE-ILD

The diagnostic criteria for ILD were the presence of bilateral reticulation and consolidation or ground-glass attenuation on high-resolution computed tomography (HRCT). The HRCT pattern was classified according to the American Thoracic Society, European Respiratory Society, Japanese Respiratory Society, and Latin American Thoracic Association (ATS/ERS/JRS/ALAT) clinical practice guideline of IPF. It was classified as usual interstitial pneumonia (UIP) pattern, probable UIP pattern, indeterminate UIP pattern, and alternative diagnosis^20^.

Postoperative AE-ILD caused by lung resection was defined based on the criteria proposed by previous studies for the disease and the ATS/ERS/JRS/ALAT statement as follows: (i) acute worsening or development of dyspnea and oxygen desaturation; (ii) CT showing new ground-glass abnormality bilaterally and/or consolidation; (iii) no evidence of other alternative causes, such as pulmonary infection, cardiac failure, pulmonary embolism, and pneumothorax; and (iv) development of AE-ILD within 30 days after lung resection^21–23^.

### Measurement of serum HMGB1 concentrations

Serum samples were collected before surgery at our hospital and stored at − 80 °C. Serum levels of HMGB1 were measured using commercially available enzyme-linked immunosorbent assay kits according to the manufacturer’s instructions (HMGB1 ELISA Kit II [Shino-Test Corporation, Tokyo]).

### Statistical analysis

Values are expressed as mean ± standard deviation or median (IQR) according to their distribution. Normally and non-normally distributed variables were evaluated using the t-test and Mann–Whitney *U* test, respectively. Pearson’s chi-squared tests were also conducted. Linear regression analysis was conducted to study the independent effects of baseline characteristics on serum HMGB1 levels. Logistic regression analysis was conducted to identify independent risk factors for the development of postoperative AE-ILD and to estimate the respective odds ratios and their 95% confidence intervals (CI). Confounders with a p-value < 0.05 according to the univariate analysis were included in the multivariate analysis. ROC curve analysis was performed to identify the optimal cut-off levels of serum HMGB1, operative time, and bleeding volume for predicting the development of postoperative AE-ILD. All statistical analyses were performed using JMP version 14.1.0 (SAS Institute Inc., Cary, NC, USA). A *P*-value of less than 0.05 was considered statistically significant.

## Abbreviations

AE: Acute exacerbation
ALAT: Latin American Thoracic Association
ATS: American Thoracic Society
AUC: Area under the curve
BALF: Bronchoalveolar lavage fluid
CEA: Carcinoembryonic antigen
CI: Confidence interval
DLco: Diffusing capacity for carbon monoxide
ERS: European Respiratory Society
FDG: Fluorodeoxyglucose
FEV1: Forced expiratory volume in one second
FVC: Forced vital capacity
HMGB1: High-mobility group box 1
HRCT: High-resolution computed tomography
ILD: Interstitial lung disease
IPF: Idiopathic pulmonary fibrosis
IQR: Interquartile range
JRS: Japanese Respiratory Society
KL-6: Krebs von den Lungen 6
OR: Odds ratio
RAGE: Receptor for advanced glycation end product
ROC: Receiver operating characteristic
SUV: Standard uptake value
TLR: Toll-like receptor
UIP: Usual interstitial pneumonia
VC: Vital capacity
